# Learning across the UK: a review of public health systems and policy approaches to early child development since political devolution

**DOI:** 10.1093/pubmed/fdz012

**Published:** 2019-03-05

**Authors:** Michelle Black, Amy Barnes, Susan Baxter, Claire Beynon, Mark Clowes, Mary Dallat, Alisha R Davies, Andrew Furber, Elizabeth Goyder, Catherine Jeffery, Evangelos I Kritsotakis, Mark Strong

**Affiliations:** 1 School of Health and Related Research, The University of Sheffield, Regent Court, 30 Regent Street, Sheffield S1 4DA, UK; 2 Public Health Wales, 2 Capital Quarter, Tyndall Street, Cardiff CF10 4BZ, UK; 3 Public Health Agency Northern Ireland, Linenhall Street Unit, 12-22 Linenhall Street, Belfast BT2 8BS, UK; 4 Division of Population Medicine, Cardiff University, Cardiff CF14 4XN, UK; 5 Public Health England (Yorkshire and the Humber), Blenheim House, Duncombe Street, Leeds LS1 4PL, UK; 6 NHS Borders, Borders General Hospital, Melrose, Roxburghshire TD69BS, UK

**Keywords:** child development, devolution, early years, policy, public health systems, systematic review

## Abstract

**Background:**

Giving children the best start in life is critical for their future health and wellbeing. Political devolution in the UK provides a natural experiment to explore how public health systems contribute to children’s early developmental outcomes across four countries.

**Method:**

A systematic literature review and input from a stakeholder group was used to develop a public health systems framework. This framework then informed analysis of public health policy approaches to early child development.

**Results:**

A total of 118 studies met the inclusion criteria. All national policies championed a ‘prevention approach’ to early child development. Political factors shaped divergence, with variation in national conceptualizations of child development (‘preparing for life’ versus ‘preparing for school’) and pre-school provision (‘universal entitlement’ or ‘earned benefit’). Poverty and resourcing were identified as key system factors that influenced outcomes. Scotland and Wales have enacted distinctive legislation focusing on wider determinants. However, this is limited by the extent of devolved powers.

**Conclusion:**

The systems framework clarifies policy complexity relating to early child development. The divergence of child development policies in the four countries and, particularly, the explicit recognition in Scottish and Welsh policy of wider determinants, creates scope for this topic to be a tracer area to compare UK public health systems longer term.

## Introduction

Giving children the best start in life is critical for public health given that early years experiences and circumstances shape lifelong health and health inequalities.^1^ Policy action to improve early developmental outcomes therefore makes social and economic sense.^2^ Yet all UK countries face challenges in improving children’s developmental outcomes in the early years and during transitions into school.^3^ Children from deprived backgrounds continue to have poorer health and education outcomes compared to the most affluent, and one in five are estimated to live in relative poverty.^3^ With increasing emphasis on a ‘prevention approach’,^4^ the public health community need to better understand how policy and the public health system contribute to children’s early developmental outcomes, so as to reflect on how to effect change. Political devolution in the UK offers a ‘natural experiment’ to learn from and identify examples of good practice.^5^

Political devolution refers to an ongoing process, initiated in 1999 under the Labour Blair Government, whereby political powers are transferred from the UK Parliament in Westminster to the Scottish Parliament, National Assembly for Wales and Northern Ireland Assembly; thereby enabling each country to exert more control over the direction of national policy.^6^ While responsibilities for key policy areas for early child development (health, education) are devolved to Scotland, Wales and Northern Ireland, devolved institutions do not have full powers over certain wider determinants of child development (macro-economics, welfare); with responsibility largely remaining in Westminster.^6^ This is pertinent for the public health community and its role in early years’ policy, not only presenting a challenge to effecting change, but also bringing to the fore the need to understand public health as a complex system.^7^ This means conceptualizing early child development as the outcome of ‘a multitude of interdependent elements within a connected whole… [which] affect each other in sometimes subtle ways, [and] with changes potentially reverberating throughout the system’.^8^ Little comparative work has been published, however, about how the UK public health systems operate in relation to child development in the early years, nor about variations in policy approaches in each country since devolution.

To address this gap, we completed a systematic review of academic and grey literature on the public health system and policy approaches to early child development in each UK country since political devolution. The review had two aims: (i) to understand policy and system approaches in each country since devolution; and (ii) to identify examples of similarities and differences across the UK systems, so as to promote learning and cross-country dialogue on how to effect change.

## Method

A systematic literature review on policy and system approaches to child development in the early years was conducted, with participatory input from an expert stakeholder group. This work was part of a wider study on public health in the four countries of the UK, which included development of a public health systems framework.^9^ The development of this framework is discussed in more detail below. The stakeholder group identified ‘school readiness’ as a key public health concern that should form the additional focal topic for review. As ‘school readiness’ is ill-defined and its meaning contested, the scope was redefined as child development in the early years, in order both to operationalize it for systematic review and avoid imposition of any particular set of values or beliefs.

Prior to starting the review, a protocol was produced setting out aims and objectives, criteria relating to inclusion and exclusion, and details of methods to be employed. The intention was not to produce a comprehensive review of all possible sources relating to child development in the early years, but rather to review literature which related more synoptically to elements of the system in the four countries of the UK and to draw out examples as a basis for future cross-country discussion about systems strengthening.

The Preferred Reporting Items for Reporting Systematic Reviews and Meta-analyses (PRISMA) guidelines were used.^10^

### Search strategy

Medline, PsycINFO and ProQuest social science electronic databases were searched in July–August 2017. Government websites were also searched in each country for details of key public policy initiatives. The stakeholder group was contacted to identify relevant grey literature. Citation searching of key authors and papers, and reference checking was also carried out. Details of the search strategy are provided in additional file 1. The full search strategy is available from the authors.

### Inclusion criteria

Studies or documents relating to policy and system approaches to address development in children up to the age of seven in England, Scotland, Wales, Northern Ireland and the UK as a whole were included. The age of seven was used to take account of Scotland’s older school entry and to be as inclusive as possible. Policy and system approaches were defined as: policies, interventions, indicators and outcomes that contribute to supporting child development in the early years. Outcomes were defined as any population-level health and wellbeing outcomes. There was no restriction on study design as we anticipated that the literature would include discussion articles, policy documents and informal evaluations. Documents published since 1999 (the year devolution commenced) were included.

### Study selection and screening

Titles and abstracts (where available) of retrieved citations were screened by a reviewer against the inclusion criteria. Any queries regarding inclusion were discussed by the research team. All citations were second checked by the principal investigator.

During the screening process we used a two-stage approach, with initial flagging of possible sources of evidence for inclusion. These documents were then discussed by the team to identify those that would be taken forward for full document review. Evidence excluded at full document review, together with the reason for exclusion, were recorded and provided in additional file 2.

### Data extraction and synthesis

Documents which met the inclusion criteria were read in full and a data extraction for each was completed. A data extraction form was developed using previous expertise of the team, and trialled on a sample of different sources. The extraction form collected data on: first author/year; study design; study participants; contextual factors; policy area; reported outcomes and impacts; processes and ways of working; influencing factors; summary of findings; and main author conclusions. We used narrative methods to synthesize the identified literature, together with the public health systems framework that we had developed in the wider project, to examine relationships between elements of public health.

### Development of the public health systems framework

An initial workshop with stakeholders from each country of the UK was convened to develop a ‘start model’ outlining key elements of a public health system. We discussed intended public health outcomes and impacts, public health activities, and factors influencing these activities and outcomes in the four countries. This start model formed the first iteration of the public health systems framework, which was then revised and refined through analysis of the included literature. We noted where elements of the start model were not reported in the literature and where there may be associations and relationships between elements. Versions of draft frameworks were returned to stakeholders following the literature review for further input, in a process of continual revision prior to production of the final version.

### Quality appraisal

Given the anticipated predominance of non-empirical studies, quality appraisal using standard tools was not considered appropriate. Our approach to quality appraisal was therefore based on the hierarchy of evidence, highlighting in the synthesis where included studies reported data, rather than author opinion.

Data were synthesized via tabulation, in addition to narrative summary and use of the public health systems framework. We focussed the synthesis on data relating to examples of similarities and differences between countries, outlining where comparative evaluations were reported.

## Results

**Fig. 1:**
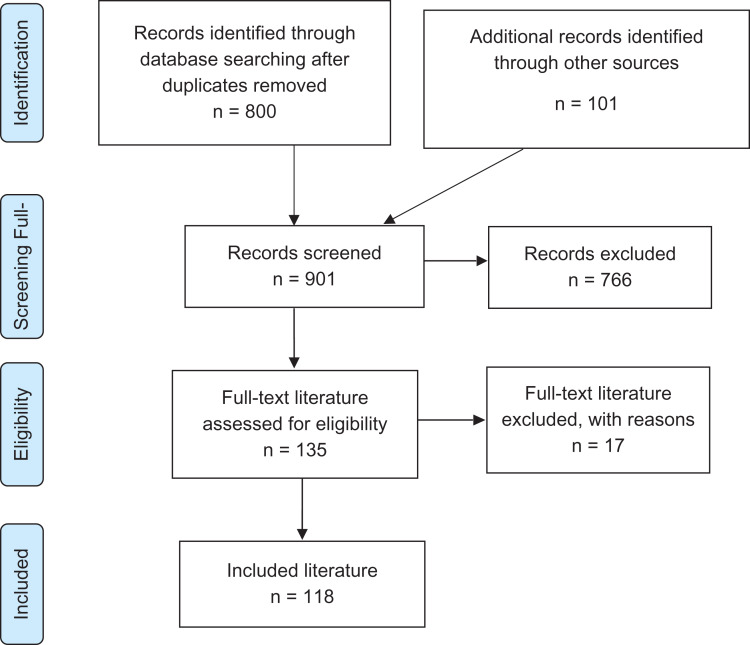
PRISMA diagram illustrating the process of literature selection.

From a database of 901 citations, 118 documents met our inclusion criteria. Figure 1 illustrates the numbers of citations included and excluded at each stage of the selection process.

### Study characteristics

Of the included 118 documents, 39 were peer reviewed journal articles,^11–49^ 39 non-peer reviewed reports (evaluation, research, audit or statistical in nature),^50–88^ 32 policy, legal or guidance documents^89–120^ and eight classified as ‘other’ (for example, briefings).^121–128^ The identified evidence sources were published between 2002 and 2017, with most published in 2013 or later (*n* = 84). We identified seven sources that had some form of comparative element: five sources^16,27,41,46,83^ compared all countries of the UK; one source^47^ focused on three countries (England, Scotland and Wales); one source^70^ on England and Scotland. Other identified sources contained evidence relating to just one of the four UK countries (*n* = 111).

Table 1 shows differences in the type of source and country of focus of the included documents. For England, the majority of the ‘single country’ evidence were journal articles (*n* = 22). For Northern Ireland, Scotland and Wales, there was a mix; with more non-peer-reviewed reports identified as relevant, alongside primary policy documentation. Summary details for each included evidence source in the review are provided in additional file 3.

**Table 1:** The type of source and country of focus of included evidence

	Type of evidence				
Country of focus	Peer-reviewed journal articles	Non-peer reviewed reports (evaluation, research, audit, statistical)	Policy, legal or guidance documents	Other (e.g. briefing note)	Totals
England	22	9	7	1	39
Northern Ireland	3	8	4	2	17
Scotland	4	14	9	3	30
Wales	5	6	12	2	25
UK	4	1	0	0	5
England, Scotland and Wales	1	0	0	0	1
England and Scotland	0	1	0	0	1
Totals	39	39	32	8	118

### Quality of studies

As outlined in the methods, it was not appropriate to use standard tools to appraise literature that was based on author opinion and description, or that were policy, legal or guidance documents. We sought to indicate in the synthesis where evidence was based on empirical work, and where there may be particular concerns regarding views or experiences expressed, or where reported findings may be of limited relevance to current contexts.

### Synthesis of results

**Figure 2:**
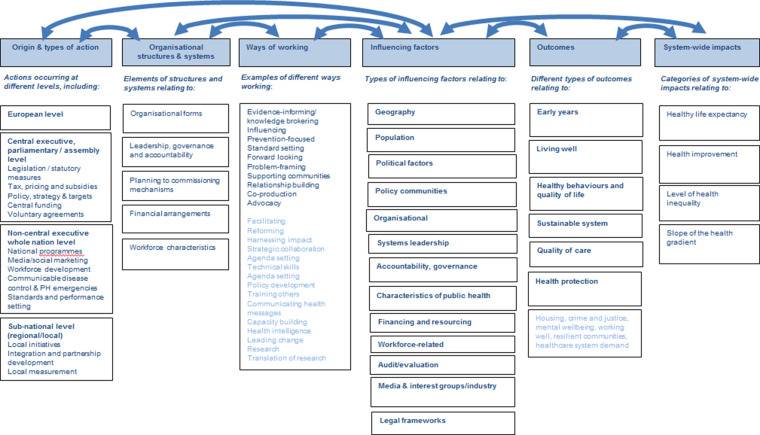
The public health systems framework. (Items in pale are those suggested at the workshop but not reported within the literature. Arrows show associations between elements.)

The initial framework developed at the workshop was a starting point for analysis, with this further developed and refined iteratively during the synthesis with input from the team and stakeholders. The final version is illustrated in Fig. 2. Data relating to each element of the system were outlined in turn (i.e. relating to each column in Fig. 2) and we have highlighted where examples of similarities and differences between the countries were identified. Infographic versions of the public health systems framework and early years policy across the UK, produced from this research, are also available.^129,130^

## Results of included studies

### Origin and types of policy action

There has been a rapid growth in early years policy action in all countries since devolution, with Scotland and Wales taking an approach that seeks to focus on wider determinants of child health.^17,18,20,23,47,48,50,55,74,77,80,99,101,107,111,112,114,120,125,127^ This is evidenced by distinctive recent legislation identified in Scotland and Wales which shapes the context for action. For example the Children and Young People (Scotland) Act 2014 which enshrined Scotland’s Getting it Right for Every Child (GIRFEC) approach in law^17,20,108^ and in Wales duties related to children’s play placed on Welsh local authorities, Wellbeing of Future Generations (Wales) Act 2015.^89,115,118,127^ English policy underpinned by a focus on competition, markets and choice was identified as diverging from other countries, as highlighted by a differing, more market-based approach to commissioning early education, learning and/or childcare and health visiting services.^27,35,46–48,54,70,75,78^

Some similarities were identified across all countries in relation to policy, with each supporting: an early intervention focus, cross-sectoral approach, play-based early years curriculum, entitlements to early education/care, integrated forms of family support and child health/parenting programmes.^11,28,35,42,44^ In addition, all countries have adopted child health programmes that are broadly similar.^97,99,100,116,120^ Yet distinctive tools or approaches for measuring and supporting child development were identified such as: the move to integrated reviews between education and health practitioners in England^99^ and Northern Ireland (for 2–2.5 year and 3+ reviews, respectively).^78,120,127^ (Table 2).

**Table 2:** Comparison of specific elements of the child health programmes in the four nations of the UK. (Sources: Refs. ^97,99,100,106,110,116,120^)

Elements of universal child programmes	England	Northern Ireland	Scotland	Wales
Programme details	Healthy Child Programme 0–19 to 0–5 years element for pregnancy and first 5 years of life	Healthy Child, Healthy Future (HCHF) Programme—pre-school element is 0–4.5 years	Child Health Programme—Health Visiting Pathway is pre-birth to pre-school	Healthy Child Wales Programme—for all families with 0–7-year-old children (Flying Start also offers an enhanced health visiting service to families/children under 4 in most deprived areas of Wales)
Universal element	Yes	Yes	Yes	Yes
Enhanced provision for families with identified needs	Yes	Yes	Yes	Yes
Scheduled Universal contacts	5 (mandated) Antenatal review Within 14 days 6–8 weeks 1 year (9–12 months) 2–2.5 years (integrated review)	9 Antenatal review 10–14 days 6–8 weeks 14–16 weeks 7–9 months 1 year 2–2.5 years 3+ review (NEW) 4–4.5 years	11 Antenatal review 11–14 days 3–5 weeks 6–8 Weeks 3 months 4 months 6 months 8 months 13–15 months 27–30 months (NEW in 2013) 4–5 years	8 Antenatal review 14 days 8, 12, 16 weeks 6 months 15 months 27 months 3.5 years 4/5 years (handover from health visitor to school nurse)
Integrated reviews between health and education sectors	Yes—health and education practitioners are working together for the 2–2.5 years review	Yes—health and pre-school education practitioners are working together to pilot a 3+ health review	Not in place	Not in place
Practical assessment and measurement tools	ASQ-3	ASQ: SE-2 (as part of 3+ review)	ASQ-3 (nationally recommended for all reviews) Other tools can be used based on professional judgements. Other recommended questionnaires: Parents Evaluation of Developmental Status (PEDS); ASQ SE-2; Strengths and Difficulties Questionnaire (SDQ); Schedule of Growing Skills II (SOGS II) National Practise Model/Wellbeing Wheel with SHANARRI indicators (Safe, Healthy, Achieving, Nurtured, Active, Respected, Responsible, Included)	Integrated tool—all Wales Health Visiting Family Resilience Assessment Instrument Tool (FRAIT) Schedule of Growing Skills (SOGS)—selective use Foundation Stage Profile Assessments

Notes: ASQ-3 = Ages and Stages Questionnaire Third Edition; ASQ:SE-2 = Ages and Stages Questionnaire:Social Emotional, Second Edition.

Divergence was identified in relation to national conceptualizations of early child development, with a distinctive variation in framing: ‘preparing for life’ versus ‘preparing for school’. Scotland was highlighted as aspiring to achieve a coherent approach focusing on the former,^17,20,50,53,107,108^ with English (and to some extent Welsh policy)^40,110,111^ more focused on ‘preparing for school’. This latter approach was criticized by some authors as being deficit-focused (what children cannot do/who is ‘left behind’), risking over-assessment, and negative labelling at an early age that could undermine future learning and development.^11,14,15,19,22,34,37,47,48^ Different policy framings were further reflected in differential approaches to pre-school provision, with pre-school provision presented as a universal commitment to education in Northern Ireland,^94,127^ or early learning and childcare in Scotland^53,105^ whilst, in part, an ‘earned’ entitlement for working parents in England and Wales.^71,98,112,114,126^

### Organizational structures and systems

Included sources highlighted a complex organizational landscape for early education and care in all countries of the UK, with, for example, a mixed economy of service provision across the public, private and voluntary sectors in all countries (i.e. nurseries, childminders, nursery classes, play groups and so on).^20,31,53,54,65,70,74^ In relation to each country, complicated forms of leadership for early child development were identified;^12,17,47,48,68,70,71,75,127^ formal partnership bodies at different levels of the system;^18,49,66,80,118,128^ and a diverse workforce for supporting early child development was highlighted.^12,46–48,53,54,66,70,80,81,106,118,125^ None of the included sources however considered these topics in detail. In all countries, the vital system role played by health visitors in supporting families and helping to improve early years child development outcomes was highlighted.^71,75,106,116,125^ Included sources contained limited detail about workforce numbers and so detailed comparisons were not possible in the context of the review. While some papers referred to health visitor numbers and caseloads,^18,65,75,116,119,125^ the included data were not directly comparable.

### Ways of working

The initial framework that was produced with expert stakeholders identified a list of descriptors that were perceived to characterize key ways of public health working. This list provided a starting point for synthesizing data relating to ways of working reported in included evidence. This was challenging as the descriptors frequently overlapped, and references to ways of working were often vague and lacked clarity. A number of included sources mentioned providing a prevention-focus (early intervention);^18,23,55,77,99,101,107,112,114,125^; co-producing;^101,116^ and asset-based or community development approaches;^13,35,69,73^, but these mostly touched on aspirations to work in particular ways, with limited detail in included sources about how or whether these would be or were being achieved. There appeared, however, to be particular emphasis on partnership working within included sources given the cross-cutting nature of early years policy action.^13,18,35,54,57,65,66,73,80,90,100,105,116^

### Influencing factors

An extensive range of influencing factors were identified within the public health systems of the UK, relating to: population and geography; political factors; financing and resourcing; workforce-related; organizational and leadership; the nature of public health; and audit, data and evaluation. These factors help explain similarities and differences in policy action in each country, organizational structures and systems, and ways of working, which, in turn, may influence outcomes and impacts; thus illustrating the complexity of action to improve early child development in the UK.

Population characteristics such as geography of disadvantage, poverty and living conditions were highlighted as fundamentally influencing early child development and policy processes in all countries.^14,15,18,20,35,49,50,53,55,61,62,64,65,67,69,73,81,84,112,125^ A range of other factors, such as parental knowledge/attitudes and home learning environment, were also highlighted as influential.^31,32,52,61,67,73,86^

Many political influences were highlighted in England, Scotland and Wales, with limited evidence on political factors in Northern Ireland. There was limited comparative evidence describing the impact of political forces for change over time across the four countries. However, political factors generated differences between countries. For example, varying levels of trust in local government were identified as significant, alongside differing political ideas about appropriate roles of the state, markets and individuals in supporting early child development across the four countries.^11,17,23,40,42,46–49,70,128^ One consequence highlighted was that the role played by central and local government varied by jurisdiction, with local authorities in Scotland and Wales having more autonomy than in England, where central government exerts more control over funding and regulation; thus limiting local authority intervention in early education/childcare.^23,46,53,54,70^ In contrast, scope for system-wide policy impact on determinants of early child development in devolved countries was highlighted as limited because key areas (welfare/social security, employment), which are inter-connected to people’s living conditions and life opportunities have largely remained reserved matters, with Westminster retaining control.^17,23,70,112^ This situation is changing: The Scotland Act (2016), for example, further devolved powers for tax, employment support and welfare-related benefits and further powers are being sought in Wales.

Financing and resourcing issues were highlighted as significant in included sources, often linked to political choices or policy decisions within the wider system.^31,75,81,84^ A range of financial or resourcing influencing factors within each system were identified: moves from ring-fenced to mainstream funding;^26,75,84^ the short-term nature of some funding;^68^ wider austerity policies and/or issues of financial sustainability, sometimes linked to funding cuts (e.g. to early education and child care, health visiting services or children’s centres, and particularly in England).^31,49,67,69,81,112^ There were examples of recent investments in Wales in terms of redevelopment of the early years curriculum and the Flying Start programme in the most deprived areas, involving integrated family support and enhanced health visiting services for families with children under four.^65,116^

In relation to workforce, sources highlighted the particularly influential role of health visitors in all countries in supporting child development in the early years; noting, for example, the importance of caseload in shaping contact time, engagement and relationship-building with families (with extended contact linked to benefits for the most vulnerable families).^65,81^ Included sources suggested that health visitors were under particular pressures, due, for example, to issues with recruitment and retention and/or high workloads, particularly in England and Northern Ireland.^18,65,75,78,81^ Workforce expertise and appropriate skills sets were further highlighted as an important influence and moderator of policy action;^31,35,40,44,49,53,70,71^ with, for example, traditional divides between ‘education’ and ‘childcare’ implicated in differential qualifications, status and pay across the early years workforce and in the quality of service provision.^53,70^

In terms of organizational factors and leadership, governance and accountability issues, included sources did not consider these topics in detail and none compared across the four countries. The significance of national leaders in advocating for early years policy action^40,48^ and local champions in shaping implementation were however highlighted;^43,85^ as well as the powerful role of the Office for Standards in Education, Children’s Services and Skills (OFSTED) in influencing policy and practice in England through its inspection regime.^34^ Spatial variability in the quality or availability of child health programmes, integrated forms of family support and early education/childcare were also highlighted;^49,53,67,71,79^ as well as variability by provider type; with provision noted as better in maintained/statutory settings in England, Northern Ireland and Scotland (sources did not include information about Wales).^35,53,74^ The significance of organizational relationships (i.e. partnerships) within the early years system was highlighted (e.g. shaping trust, communication, coordination, data sharing);^17,73,80^ with pre-school to school relationships particularly influential in shaping transition experiences into school and moderating the extent to which children started school with any disadvantages.^11,69,71^

### Outcomes and system wide impacts

Difficulties in monitoring and evaluating complex policy action to support early child development within complex public health systems of the four countries were highlighted. This included challenges in demonstrating and attributing the effects of particular policies when there are long time-lags and many intervening factors between action and impact, and limitations associated with information systems/data availability.^13,23,27,28,52,61,62,65,67,69,73,80,125^ For example, recent challenges were highlighted in evaluating the impact of children’s centres in England due to their varied nature, varied patterns of family use of services, and because policy changed over time.^81^ Continuing developmental inequalities between children of differing socio-economic status were noted across all countries.^14,15,27,32,40,50,67,71,83^ Against this background, included sources did not highlight any ‘success’ story—no system was uniformly ‘better’ or ‘worse’—with emphasis in one source that any judgement depends on what measures of child development are considered and how (i.e. at what levels of comparison).^41^ England, Scotland and Northern Ireland all capture Ages and Stages Questionnaire (ASQ) data as part of child health programmes, but no country comparisons were found in the review.

## Discussion

### Main finding of this study

Our aim was to explore policy and systems in relation to child development in the early years since political devolution and to identify examples of similarities and differences across the four countries of the UK. Whilst there was a paucity of literature directly comparing the four countries, the scope of our review allowed us to describe the policy approaches across the countries. Our main finding is that there is variation across the countries which is both interesting and an opportunity for learning and action. It is clear that child development in the early years is identified as a key ‘prevention approach’ to public health in each UK country. There is much policy rhetoric around the importance of this area to public health and there has been a growth in early years related action in each country. Our findings suggest however, that policy action to support child development is part of a complex systems landscape in all of the UK countries and that this system is subject to many pressures and influences. A range of policy action was identified in all countries, at different levels. Together, this contributes to and shapes children’s early developmental outcomes,^20^ but there are many potential pathways in the relationship between policy action (interventions/inputs), outcomes and impacts, as well as many potential moderators (influencing factors) of that relationship.

Across all countries, poverty and resourcing issues were identified as key influencing factors, as well as the significance of inter-organizational relationships (partnerships). Particular challenges were identified in England and Northern Ireland, including, for example: pressures relating to short-term funding or funding cuts to children’s centres, issues of financial sustainability in relation to early education or childcare, and pressures on health visitors who are recognized as key members of the early years public health workforce. Yet some of these issues were also mentioned in relation to Scotland and Wales. Examples of positive developments were identified in England and Northern Ireland, such as the move to integrated reviews between health and education practitioners in the early years (at 2–2.5 and 3+ years, respectively), from which there could be valuable opportunities for wider learning. Political factors shaped divergence, with variation in national conceptualizations of child development (‘preparing for life’ in Scotland versus ‘preparing for school’ in England and Wales) and pre-school provision which is a ‘universal entitlement’ in Northern Ireland and Scotland but, in part, an ‘earned benefit’ for working parents in England and Wales. Understanding how these differences in policy approaches impact on child development outcomes is of interest. Our findings suggest that the legislative and policy context for early years may be more positive in Scotland and Wales in public health terms, where distinctive legislation focusing on wider determinants has been progressed. However, this is limited by the extent of devolved powers.

Across all countries, continuing developmental inequalities for children in the early years were reported according to socio-economic status. This highlights the need for continued policy action to give children the best start in life across the UK as a means to help address this key wider determinant of health and health inequalities. It is not possible to determine whether any country is uniformly better or worse in this regard due to the varying measures used and the difficulties of attributing change in a complex system. Recognizing continuing challenges—not least in terms of evaluating large-scale early years initiatives and programmes—there are opportunities for shared learning between countries. However, without a more robust evidence base and systems-based evaluation mechanisms for assessing the impact of early years policy there would appear, currently, to be a precarious basis on which to form decisions about whether to continue particular forms of action. This highlights a challenge to the public health workforce.

There are opportunities for further research, particularly in relation to learning from the moves towards integrated health and education reviews in England and Northern Ireland, as well as the recent shifts in the legislative and policy context in Scotland and Wales. We suggest that it would be useful to unpick the elements of the system that have led to the broader policy changes identified and to track how these reshape (or not) the wider public health system. For example, appraising, in more detail, how differing political ideas within the four countries, about the appropriate roles of the state, market and individuals in early years provision, shifting devolved powers, relationships between central and local government and also the role of public health actors are contributing and interacting in the shaping of children’s developmental outcomes.^8^ More research is also needed on the relevance and weight of the influencing factors identified in the review, particularly political factors and the political forces for change. In addition, there is a need for improved understanding and discussion across the four countries about how influences relating to funding and the contribution of public health policy in shaping outcomes. In particular, further research and methods are needed to evaluate policy in the natural experiment contexts provided by the devolution agenda. In summary, we suggest, more broadly, that the whole system relating to child development in the early years could usefully be followed up as a ‘tracer’ area of a prevention approach to public health, and thus as a key area for future systems comparison, learning and dialogue across the four UK countries.

### What is already known on this topic

Giving children the best start in life is an important public health priority, yet inequality in children’s developmental outcomes persist as childhood poverty continues to increase.^131^ It is recognized that further research is needed on the interaction between different policy approaches and the determinants of child development, long-term health and health inequalities in order to prioritize policies that are likely to have the greatest impact.^131^ There is limited published evidence on how the UK public health systems operate in relation to child development in the early years, and on variations in policy approaches in each country since political devolution.

### What this study adds

This study describes how public health policy in relation to early child development in the four countries of the UK compares since political devolution. It highlights the complexity and challenges of this policy area and how system wide understanding and change is required for impact. The public health workforce has an opportunity to maximize effectiveness by adopting a systems approach and exercising influencing skills and partnership working, particularly at central executive level.

The devolved countries face challenges in tackling determinants, as there are limits to the extent of devolution in the areas of welfare provision, employment support and macro-economics. This hinders their ability to redress poverty, one of the main influencing factors for children’s outcomes across the life course. Persistent developmental inequalities between children in differing socio-economic status in all countries highlights a critical need for continued action, across the wider determinants, to give children the best start in life across the UK.

Our study highlights the challenges in tracking system change and impact and the opportunity to develop our understanding by tracing the prevention approach taken in Scotland and Wales given the focus on wider determinants, as compared with England.

The public health systems framework developed in this work can be a useful basis for future dialogue and reflection about public health from a systems perspective, and to facilitate future systems-based evaluations. The infographics produced from this research may help in this regard.^129,130^

### Limitations of this study

The focus of the review was defined as ‘child development in the early years’. It therefore only explores examples of one subsection of a wider field of healthy child development and early years work (other issues would include healthy weight, breastfeeding, immunization and potentially many other areas of public health activity). In addition, it is a policy area undergoing significant change. As such, findings from currently published sources may be limited by the fact they have been superseded by recent policy developments.

As our approach was to explore similarities and differences in public health policy and systems, rather than public health outcomes, our study is limited to outcomes found within child public health policy literature. Therefore, rather than to judge, our approach was to support a broader discussion about whether policy action in the early years has been reshaping the whole systems in favourable, health-promoting ways.^8^

## Conclusion

Early child development is on the policy agenda in each UK country, but public health systems are subject to many influences, which shape outcomes. The public health systems framework is an aid to improve understanding of policy complexity relating to early child development and can promote dialogue to facilitate system change to improve outcomes. The divergence of child development policies in the four countries and in particular the explicit recognition in Scottish and Welsh policy of wider determinants, creates scope for this topic to be a tracer area to compare UK public health systems in the longer term.

## Supplementary Material

fdz012_Additional_file_1_-_search_terms

fdz012_Additional_file_2revised_-_excluded_papers

fdz012_Additional_file_3_-_included_papers
