# Supplementary material for: Learning across the UK: a review of public health systems and policy approaches to early child development since political devolution
Source: J Public Health (Oxf). 2019 Mar 5;42(2):224–38. doi: 10.1093/pubmed/fdz012 (PMC7251421; doi:10.1093/pubmed/fdz012)
Supplement: fdz012_Additional_file_1_-_search_terms [file fdz012_additional_file_1_-_search_terms.docx]

**Additional file 1. Terms included in the search strategy**

| **School readiness**  School adj (ready or readiness)  Achievement gap  Attainment gap  (Barriers to) learning  Learning (outcomes)  Academic outcomes  Cognition  Cognitive development  Motor development  Language development  Reading  Literacy  Numeracy  Behaviour  Toilet training  Transition | **Age group**  Infant  Early years  Pre-school  Playgroup / play group  Nursery  Sure start  Primary school*  P1 | **Policies and interventions**  Policy / policies  Devol*  Legislat*  Regulat*  Govern*  Law*  Intervention*  Curriculum for Excellence  Attainment Challenge  **Measures**  Scales  Measur*  Assess*  Indicator*  Test*  ASQ (“Ages and stages” questionnaire)  Growing Up In Scotland  GIRFEC (Getting it right for every child)  SDQ (Strengths & Difficulties questionnaire) | **UK**  United Kingdom  Brit*  Scotland  Wales  (Northern) Ireland  London  Westminster  Holyrood  Belfast  Cardiff  Edinburgh  Glasgow  Highlands and Islands |
| --- | --- | --- | --- |
