# Supplementary material for: Learning across the UK: a review of public health systems and policy approaches to early child development since political devolution
Source: J Public Health (Oxf). 2019 Mar 5;42(2):224–38. doi: 10.1093/pubmed/fdz012 (PMC7251421; doi:10.1093/pubmed/fdz012)
Supplement: fdz012_Additional_file_3_-_included_papers [file fdz012_additional_file_3_-_included_papers.docx]

**Additional file 3: Summary of included sources in the school readiness review.**

| **First author, date, source title** | **Source Type** | **Design** | **Country / Countries** |
| --- | --- | --- | --- |
| Ang (2014). Preschool or Prep School? Rethinking the Role of Early Years Education. | journal article | Descriptive | England |
| Belsky J *et al.* (2006) Effects of Sure Start Local Programmes on children and families: early findings from a quasi-experimental, cross sectional study. | journal article | Descriptive | England |
| Belsky J *et al.* (2008). Research and Policy in Developing an Early Years’ Initiative: The Case of Sure Start. | journal article | Descriptive | England |
| Bradbury, A. (2013). Education policy and the 'ideal learner': producing recognisable learner- subjects through early years assessment. | journal article | Qualitative | England |
| Bradbury, A. (2011). Rethinking assessment and inequality: The production of disparities in attainment in early years education. | journal article | Qualitative | England |
| Bradshaw P *et al.* (2015). Growing Up in Scotland: The Circumstance and Experiences of 3 year olds living in Scotland 2007/2008 and 2013. | Evaluation / research / audit/ statistical report | Quantitative | Scotland |
| Bradshaw, P. *et al.* (2012). Growing Up in Scotland: Early experiences of Primary School. | Evaluation / research / audit/ statistical report | Quantitative | Scotland |
| Bradshaw *et al.* (2016) Language Development and Enjoyment of Reading: Impacts of early parent-child activities in two growing up in Scotland cohorts. | Evaluation / research / audit/ statistical report | Quantitative | Scotland |
| Bywater T. and Sharples, J. (2014). Effective evidence-based interventions for emotional well-being: Lessons for policy and practice. | journal article | Review | All UK |
| Care Inspectorate (2016a) Scotland’s early learning and childcare – an initial overview of the expansion of provision during 2014/15. | Evaluation / research / audit/ statistical report | Descriptive | Scotland |
| Care Inspectorate (2016b) Review of Scotland’s Early Learning and Childcare Expansion. | Evaluation / research / audit/ statistical report | Mixed methods | Scotland |
| Centre for Effective Services (2013). Prevention and early intervention in children and young people's services - Child health and development. | Evaluation / research / audit/ statistical report | Descriptive | Northern Ireland |
| Children and Families (Wales) Measure (2010) | Policy/legal/guidance document | Descriptive | Wales |
| Children in Wales (2012). Legislative framework for Childcare in Wales. | Other - briefing note | Descriptive | Wales |
| CNP (2017). Colin Neighbourhood Partnership. | Other - briefing note | Descriptive | Northern Ireland |
| Coles E. *et al.* (2016). Getting It Right for Every Child: A National Policy Framework to Promote Children's Well-being in Scotland, United Kingdom. | journal article | Descriptive | Scotland |
| Couper, S and Mackie, P. (2016). Scottish Public Health Network (ScotPHN) 'Polishing the Diamonds' Addressing Adverse Childhood Experiences in Scotland. | Evaluation / research / audit/ statistical report | Descriptive | Scotland |
| Cowley *et al.* (2009). Too little for early interventions? Examining the policy-practice gap in English health visiting services and organization. | journal article | Quantitative | England |
| Cunningham-Burley S. *et al.* (2002). Mapping Sure Start Scotland. | Evaluation / research / audit/ statistical report | Mixed methods | Scotland |
| CYPIC (2017). Child and Young People Improvement Collaborative (CYPIC) (2017) Children and Young People Improvement Collaborative – Vision and Outcome Aims. | Policy/legal/guidance document | Descriptive | Scotland |
| Darbyshire N. *et al.* (2014). An Unsure Start for Young Children in English Urban Primary Schools. | journal article | Descriptive | England |
| Department for Education (2013) Sure Start Children’s Centres Statutory Guidance. | Policy/legal/guidance document | Descriptive | England |
| Department for Education (2017). Early education and childcare - Statutory guidance for local authorities. | Policy/legal/guidance document | Descriptive | England |
| Department of Education NI (2006). Curricular guidance for pre-school education. | Policy/legal/guidance document | Descriptive | Northern Ireland |
| Department of Education NI (2017) Important Information for Parents Applying for a Pre-school Education Place 2017/18 School Year. | Policy/legal/guidance document | Descriptive | Northern Ireland |
| Department of Health (2015) Universal Health Visitor Reviews Advice for local authorities in delivery of the mandated universal health visitor reviews from 1 October 2015. | Policy/legal/guidance document | Descriptive | England |
| Department of Health (2016) Improving Outcomes and supporting Transparency: Part2: Summary technical specifications of public health indicators. DH August 2016. | Policy/legal/guidance document | Descriptive | England |
| Department of Health, Social Services and Public Safety (2010). Healthy Child, Healthy Future - A Framework for the Universal Child Health Promotion Programme in Northern Ireland. | Policy/legal/guidance document | Descriptive | Northern Ireland |
| Dunlop A.W. (2015). Aspirations and actions: early childhood from policy to practice in Scotland. | journal article | Policy review | Scotland |
| DWP (2017) Improving lives: helping workless families. | Policy/legal/guidance document | Descriptive | England |
| East Lothian Council (2017) Early development instrument. | Other - briefing note | Descriptive | Scotland |
| Ellis, S. and Moss, G. (2014). Ethics, education policy and research: the phonics question reconsidered. | journal article | Descriptive | England |
| ETI (2016). Chief Inspector’s Report 2014-2016. Education and Training Inspectorate. | Evaluation / research / audit/ statistical report | Descriptive | Northern Ireland |
| Evans, K. (2013). School Readiness: The Struggle for Complexity. | journal article | Descriptive | England |
| Geddes R. *et al.* (2011). A rapid review of key strategies to improve the cognitive and social development of children in Scotland. | journal article | Review | Scotland |
| Glasgow Centre for Population Health (2017) SDQ - pre-school children. | Other - briefing note | Descriptive | Scotland |
| Hatcher, *et al.* (2006). Evidence for the Effectiveness of the Early Literacy Support Programme. | journal article | Quantitative | England |
| Hutchings, *et al.* (2017). Evaluating the Incredible Years Toddler Parenting Programme with parents of toddlers in disadvantaged (Flying Start) areas of Wales. | journal article | Mixed methods | Wales |
| ISD Scotland (2016) Methodology to produce a revised estimate of health visitor staff in post as at 31 March 2014 | Evaluation / research / audit/ statistical report | Descriptive | Scotland |
| ISD Scotland (2017a) Child Health 27-30 Month Review Statistics Scotland 2015/16. | Evaluation / research / audit/ statistical report | Quantitative | Scotland |
| ISD Scotland (2017b) Child Health Programme Child Health Systems Programme Pre-School (CHSP Pre-School). | Policy/legal/guidance document | Descriptive | Scotland |
| Kidner, C. (2011). SPICe Briefing - Early Years – Subject Profile. | Other - briefing note | Descriptive | Scotland |
| Knibbs *et al.* (2013). National Evaluation of Flying Start: Impact Report. | Evaluation / research / audit/ statistical report | Quantitative | Wales |
| Lea *et al.* (2015). Evaluation of Making it REAL (Raising Early Achievement in Literacy). | Evaluation / research / audit/ statistical report | Descriptive | England |
| Lewis, (2011). From Sure Start to children’s centres: an analysis of policy change in English early years programmes. | journal article | Mixed methods | England |
| Local Government Association (2016) Get in on the Act – Childcare Act 2016. | Other - briefing note | Descriptive | England |
| Machin *et al.* (2013). Educational attainment across the UK nations: Performance, inequality and evidence. | journal article | Mixed methods | All UK |
| Maryat *et al.* (2014). Parenting Support Framework Evaluation, August 2009 to December 2013 - Final Report. | Evaluation / research / audit/ statistical report | Mixed methods | Scotland |
| McCoy, E. (2011). Local Authorities Improving Life Chances: A Review of a New Approach to Raising Literacy Levels. | Evaluation / research / audit/ statistical report | Descriptive | England |
| McGuiness C. et al (2014). Impact of a play-based curriculum in the first two years of primary school: Literacy and numeracy outcomes over seven years. | journal article | Quantitative | Northern Ireland |
| Melhuish E. *et al.* (2008). National Evaluation of Sure Start Research Team. Effects of fully-established Sure Start Local Programmes on 3-year-old children and their families living in England: a quasi-experimental observational study. | journal article | Quantitative | England |
| Melhuish E. *et al.* (2007). Variation in community intervention programmes and consequences for children and families: the example of Sure Start Local Programmes. | journal article | Quantitative | England |
| Melhuish, E. (2016). Longitudinal research and early years policy development in the UK. | journal article | Descriptive | England |
| Melhuish E. *et al.* (2010). Evaluation and value of sure start. | journal article | Descriptive | England |
| Merrell C. and Tymms, P. (2011). Changes in children's cognitive development at the start of school in England 2001-2008. | journal article | Quantitative | England |
| Montgomery A. *et al.* (2016). EITP Workstream 2: An Audit of Evidence-Based Parenting Programmes in Northern Ireland. | Evaluation / research / audit/ statistical report | Mixed methods | Northern Ireland |
| Morris M. *et al.* (2014). Flying Start Synthesis Report. | Evaluation / research / audit/ statistical report | Mixed methods | Wales |
| Mulholland et al (2016). Summary of Community Planning Partnerships‟ (CPPs) Early Years Change Fund Returns (2012-2015). | Evaluation / research / audit/ statistical report | Descriptive | Scotland |
| NAO (2016). Entitlement to free early education and childcare. Report by the Comptroller and Auditor General. | Evaluation / research / audit/ statistical report | Descriptive | England |
| National Children’s Bureau (2016). Incredible Years in Northern Ireland: Mapping & Fidelity Report 2013-15. | Evaluation / research / audit/ statistical report | Mixed methods | Northern Ireland |
| National Foundation for Educational Research (2010). Evaluation of Integrated Children's Centres in Wales. | Evaluation / research / audit/ statistical report | Qualitative | Wales |
| Naumann *et al.* (2013). Early Childhood Education and Care Provision: International Review of Policy, Delivery and Funding Final Report. | Evaluation / research / audit/ statistical report | Descriptive | England and Scotland |
| Neaum S. (2016). School Readiness and Pedagogies of Competence and Performance: Theorising the Troubled Relationship between Early Years and Early Years Policy. | journal article | Descriptive | England |
| NESS team (2010). The impact of Sure Start Local Programmes on five year olds and their families. Research Report DFE-RR067. | Evaluation / research / audit/ statistical report | Quantitative | England |
| Northern Ireland Executive (2016) Draft Programme for Government Framework (PfG) 2016 – 21. | Policy/legal/guidance document | Descriptive | Northern Ireland |
| OFSTED (2015). Early years -The report of Her Majesty’s Chief Inspector of Education, Children’s Services and Skills 2015. | Evaluation / research / audit/ statistical report | Descriptive | England |
| O’Hagan, J (2017). Northern Ireland Health and Social Care Workforce Census March 2017 | Evaluation / research / audit/ statistical report | Descriptive | Northern Ireland |
| Ormston, R. *et al.* (2014). Evaluation of the family Nurse Partnership Programme in NHS Lothian, Scotland: Summary of Key Learning and Implications. | Evaluation / research / audit/ statistical report | Mixed methods | Scotland |
| Perry, C. (2013). Early years provision. NIAR 68-13. Research and Information Service - Research Paper. | Other - briefing note | Descriptive | Northern Ireland |
| Perry, C. (2016). Programme for Government: Education. Research and Information Service - Research Paper. | Evaluation / research / audit/ statistical report | Descriptive | Northern Ireland |
| PHE (2016). Review of mandation for the universal health visiting service. | Evaluation / research / audit/ statistical report | Mixed methods | England |
| PHE (2016b) Health Matters: Giving every child the best start in life. | Policy/legal/guidance document | Descriptive | England |
| PHE (2016c) Best start in life and beyond: Improving public health outcomes for children, young people and families - Guidance to support the commissioning of the Healthy Child Programme 0-19: Health Visiting and School Nursing services | Policy/legal/guidance document | Descriptive | England |
| PHW (2015) Welsh Adverse Childhood Experiences (ACE) Study - Adverse Childhood Experiences and their impact on health-harming behaviours in the Welsh adult population. | Evaluation / research / audit/ statistical report | Descriptive | Wales |
| PHW (2016) Making a Difference: Investing in Sustainable Health and Well-being for the People of Wales | Evaluation / research / audit/ statistical report | Descriptive | Wales |
| PHW (2017) Our Strategic Plan 2017-2020. | Policy/legal/guidance document | Descriptive | Wales |
| Pugh, G. (2010). Improving Outcomes for Young Children: Can We Narrow the Gap? | journal article | Descriptive | England |
| Rankin J. *et al.* (2015). Tackling Health Inequalities in Scotland: an Innovative Approach to Implement the ‘Early Years’ Policy into Practice. | journal article | Descriptive | Scotland |
| Regulation and Quality Improvement Authority (2016). Every Child Counts Regional Audit of the Child Health Promotion Programme – Health Visiting and School Nursing Service. | Evaluation / research / audit/ statistical report | Mixed methods | Northern Ireland |
| Roberts-Holmes, G. (2015). The "Datafication" of Early Years Pedagogy: "If the Teaching Is Good, the Data Should Be Good and if There's Bad Teaching, There Is Bad Data. | journal article | Qualitative | England |
| Robling *et al.* (2015) Building Blocks trial Executive Summary. Evaluating the Family Nurse Partnership in England: A Randomised Control Trial 006/0060. | Evaluation / research / audit/ statistical report | Mixed methods | England |
| Robling et al (2016). Effectiveness of a nurse-led intensive home-visitation programme for first-time teenage mothers (Building Blocks): a pragmatic randomised controlled trial. | journal article | Mixed methods | England |
| RSM McClure Watters (2015). Independent Review of the Sure Start Programme. | Evaluation / research / audit/ statistical report | Mixed methods | Northern Ireland |
| Sammons *et al.* (2015). The impact of children’s centres: studying the effects of children's centres in promoting better outcomes for young children and their families. The impact of children’s centres: studying the effects of children's centres in promoting better outcomes for young children and their families Evaluation of Children’s Centres in England (ECCE, Strand 4). | Evaluation / research / audit/ statistical report | Mixed methods | England |
| Save the Children (2017). The Children Act 2004. | Other - briefing note | Descriptive | Wales |
| ScotPHO (2017) Online Profile Tool v2.0.1. | Policy/legal/guidance document | Descriptive | Scotland |
| Scottish Government (2012a) Supporting Children’s and Young People’s Learning: A Report on Progress of Implementation of The Education (Additional Support for Learning) (Scotland) Act 2004 (As Amended). | Evaluation / research / audit/ statistical report | Descriptive | Scotland |
| Scottish Government (2014) Building the Ambition National Practice Guidance on Early Learning and Childcare Children and Young People (Scotland) Act 2014. | Policy/legal/guidance document | Descriptive | Scotland |
| Scottish Government (2008) The Early Years Framework. | Policy/legal/guidance document | Descriptive | Scotland |
| Scottish Government (2015a). OECD-Scotland Education Policy Review - A background report by the Scottish Government. | Policy/legal/guidance document | Descriptive | Scotland |
| Scottish Government (2015b) Universal Health Visiting Pathway in Scotland Pre-Birth to Pre-School. | Policy/legal/guidance document | Descriptive | Scotland |
| Scottish Government 2016 National Outcomes. | Policy/legal/guidance document | Descriptive | Scotland |
| Scottish Government, 2017 Wellbeing. | Policy/legal/guidance document | Descriptive | Scotland |
| Smith, T. (2007). Early Years services in Britain 1997‐2007: a quiet revolution? | journal article | Descriptive | England |
| Social Mobility Commission (2016) State of the Nation 2016: Social Mobility in Great Britain. | Evaluation / research / audit/ statistical report | Descriptive | All UK |
| Sylva K and Pugh G (2005). Transforming the Early Years in England. | journal article | Descriptive | England |
| Taylor C. *et al.* (2016) Implementing curriculum reform in Wales: the case of the Foundation Phase. | journal article | Mixed methods | Wales |
| Taylor C. *et al.* (2013): Devolution and geographies of education: the use of the Millennium Cohort Study for ‘home international’ comparisons across the UK. | journal article | Quantitative | All UK |
| Taylor C. *et al.* (2015). Evaluating the Impact of Early Years Educational Reform in Wales to Age Seven: The Potential Use of the UK Millennium Cohort Study. | journal article | Quantitative | Wales |
| Wainwright N. *et al.* (2016). The Foundation Phase in Wales – a play-based curriculum that supports the development of physical literacy. | journal article | Mixed methods | Wales |
| Walsh, G. *et al.* (2006). An appropriate curriculum for the 4–5 year old child in Northern Ireland: Comparing play-based and formal approaches. | journal article | Mixed methods | Northern Ireland |
| Walsh G. *et al.* (2010). Implementing a Play-Based and Developmentally Appropriate Curriculum in Northern Ireland Primary Schools: What Lessons Have We Learned? | journal article | Mixed methods | Northern Ireland |
| Welsh Government (2009). Flying Start and Cymorth: An Interim Evaluation Report | Evaluation / research / audit/ statistical report | Mixed methods | Wales |
| Welsh Government (2011). Healthy and Sustainable Pre-School Scheme National Award Criteria September 2011. | Policy/legal/guidance document | Descriptive | Wales |
| Welsh Government (2012) A vision for health visiting in Wales. | Policy/legal/guidance document | Descriptive | Wales |
| Welsh Government (2013) Building a Brighter Future: Early Years and Child Care Plan. | Policy/legal/guidance document | Descriptive | Wales |
| Welsh Government (2015a). Child Poverty Strategy for Wales. | Policy/legal/guidance document | Descriptive | Wales |
| Welsh Government (2015b) Early Years Outcomes Framework. | Policy/legal/guidance document | Descriptive | Wales |
| Welsh Government (2016a) Taking Wales Forward 2016-2021. | Policy/legal/guidance document | Descriptive | Wales |
| Welsh Government (2016b) Well-being of Future Generations (Wales) Act 2015. | Policy/legal/guidance document | Descriptive | Wales |
| Welsh Government (2016c). An overview of the Healthy Child Wales Programme. | Policy/legal/guidance document | Descriptive | Wales |
| Welsh Government (2016d) Measuring the health and wellbeing of a nation - Public Health Outcomes Framework for Wales. | Policy/legal/guidance document | Descriptive | Wales |
| Welsh Government (2016e) Shared Purpose: Shared Future Statutory guidance on the Well-being of Future Generations (Wales) Act 2015 | Policy/legal/guidance document | Descriptive | Wales |
| West A. (2015). Education policy and governance in England under the Coalition Government (2010–15): Academies, the pupil premium, and free early education. | journal article | Descriptive | All UK |
| White C. and Gibb J. (2013). Early Years Peer to Peer Support Programme Evaluation of effectiveness and impact. | Evaluation / research / audit/ statistical report | Qualitative | England |
| Wincott, D. (2005) Reshaping public space? Devolution and policy change in British early childhood education and care. | journal article | Descriptive | England, Scotland and Wales |
| Wincott, D. (2006) Devolution and the Welfare State: Lessons from Early Childhood Education and Care Policy in Wales. | journal article | Descriptive | Wales |
| Woolfson L. and King, J. (2008). Evaluation of The Extended Pre-School Provision for Vulnerable Two Year Olds Pilot Programme Final Report. | Evaluation / research / audit/ statistical report | Mixed methods | Scotland |
